# Study design and protocol of a randomized, pragmatic, comparative effectiveness trial evaluating a sequenced strategy for improving outcomes in people with knee osteoarthritis pain (SKOAP): Conservative treatment evaluation

**DOI:** 10.1016/j.semarthrit.2025.152834

**Published:** 2025-09-23

**Authors:** Heavon M. Allen, Melinda M. Holena, Lauren E. Allen, SiNing Zhao, Renan C. Castillo, Steven P. Cohen, Robert W. Hurley, Daniel O. Scharfstein, Jennifer A. Haythornthwaite, Srinivasa N. Raja, Stephen T. Wegener, Christine M. Rini, Francis J. Keefe, Jordan Bridges, Ron Reeder, Richard E. Thompson, Dan Hanley, Claudia M. Campbell, the SKOAP Consortium

**Affiliations:** a Johns Hopkins University, School of Medicine, Department of Psychiatry and Behavioral Sciences, 5510 Nathan Shock Drive, Ste 100, Baltimore, MD 21224, USA; b Johns Hopkins University, School of Public Health, Major Extremity Trauma Research Consortium, 415N. Washington Street, Baltimore, MD 21231, USA; c Northwestern University Feinberg School of Medicine, Departments of Anesthesiology, Neurology, Physical Medicine & Rehabilitation, Psychiatry and Neurological Surgery, 259 E Erie St Ste 1400, Chicago, IL 60611, USA; d Wake Forest University School of Medicine, Departments of Anesthesiology, Translational Neuroscience, and Public Health; Pain Outcomes Lab; Medical Center Blvd, Winston-Salem, NC 27157, USA; e University of Utah, School of Medicine, Department of Population Health Sciences, Division of Biostatistics, 295 Chipeta Way, Salt Lake City, UT 84108, USA; f Johns Hopkins University, School of Medicine, Department of Anesthesia and Critical Care Medicine, Division of Pain Medicine, 600N. Wolfe St., Baltimore, MD 21287, USA; g Johns Hopkins University School of Medicine, Department of Physical Medicine and Rehabilitation, 600N. Wolfe Street, Baltimore, MD 2128A7, USA; h Northwestern University Feinberg School of Medicine, Department of Medical Social Science, 625N. Michigan Avenue, Chicago, IL 60611; Robert H. Lurie Comprehensive Cancer Center of Northwestern University; 675N St Clair St Fl 21 Ste 100, Chicago, IL 60611, USA; i Duke University, School of Medicine, Departments of Psychiatry and Behavioral Sciences, Psychology and Neuroscience, Medicine, and Anesthesiology, 2200W Main St, Suite 340, Durham, NC 27705, USA; j University of Utah, Utah Data Coordinating Center, 295 Chipeta Way, Salt Lake City, UT 84108, USA; k University of Utah, School of Medicine, Department of Pediatrics, 295 Chipeta Way, Salt Lake City, UT 84108, USA; l Johns Hopkins University, School of Medicine, Johns Hopkins Institute for Clinical & Translational Research, Brian Injury Outcomes (BIOS), 750 E. Pratt St., 16th floor, Baltimore, MD 21202, USA

**Keywords:** Knee osteoarthritis, Chronic pain, Duloxetine, CBT, Cognitive behavioral therapy, Pragmatic clinical trials

## Abstract

**Background:**

Treatment guidelines for knee osteoarthritis (KOA) vary across organizations, partly due to the lack of high-quality evidence. Experts disagree on the role of psychological management, pharmacologic treatments including opioids, and interventional therapies.

**Methods/Design:**

The Sequenced strategy for Knee OsteoArthritis Pain (SKOAP) trial is a multi-site, randomized, pragmatic clinical trial that uses a two-phase sequential design to evaluate the effectiveness of several interventions in individuals reporting KOA pain. Described here is the protocol for Phase 1 of the trial sequence which focuses on conservative treatments. All participants receive Best Practices (BP), a guideline-based approach to care that includes physical therapies, alternative treatments, and over-the-counter medications. Participants are then randomized to one of three groups: (1) BP alone, (2) BP plus duloxetine (30–120 mg/day), or (3) BP plus duloxetine and painTRAINER, a web-based, Cognitive Behavioral Therapy (CBT)-informed pain coping skills training. Phase 1 aims to determine whether the combination of duloxetine and BP improves pain compared to BP alone, and whether the combination of painTRAINER, duloxetine and BP provides additional benefit compared to duloxetine combined with BP. The analysis will include a modified Intention to Treat (mITT) approach and two Per-Protocol (PP) analyses; Receipt of Prescription (PP-ROP) and Minimum Effective Dose (PP-MinED). A third aim of Phase 1 is to identify clinical characteristics, patient-level factors, and psychosocial phenotypes that predict short- and long-term outcomes.

**Discussion:**

Findings from Phase 1 of the SKOAP trial will provide evidence on the effectiveness of non-opioid pharmacologic and psychological interventions for the treatment of painful KOA beyond established best practices. It may also help refine personalized treatment strategies.

## Introduction

Knee pain is one of the leading causes of disability worldwide [1], with a lifetime prevalence rate of approximately 45 % [2]. Among the various etiologies for knee pain, osteoarthritis (OA) is by far the most common [3]. Knee osteoarthritis (KOA) is a leading cause of pain and functional disability, particularly among older adults [4]. Rates of KOA have more than doubled in the past 70 years and are expected to continue rising due to increases in life expectancy and population body mass index (BMI) [4]. The prevalence, and consequently the socioeconomic burden, of KOA increases with age, obesity, and a history of trauma [5]. Guidelines for treating KOA generally support use of non-steroidal anti-inflammatory drugs (NSAIDs), patient education, weight loss, physical therapy, and exercise as standard of care practices [6]. While core conservative treatments such as physical therapy, education, and NSAIDs are widely recommended, current guidelines provide limited implementation guidance when multiple treatments are used concurrently or tailored to individual patients [7]. Most guidelines offer limited direction on combining or sequencing multiple therapies and emphasize the need for research to determine which treatments—or combinations—provide the greatest benefit for specific patient subgroups.

Over 600,000 knee replacement surgeries are performed annually for refractory KOA, with the number expected to increase [8]. The main indication for knee surgery is moderate-to-severe pain accompanied by functional limitation and physical disability. Observational data suggest that the alleviation of pain may prevent or postpone surgery [9]. Although many patients obtain meaningful relief from joint replacement, it is not always accessible or effective for all patients; it also carries increased cost and risk compared to less invasive treatments, and typically only lasts between 15 and 25 years [10]. Thus, there is a need for non-surgical treatment approaches.

Stand-alone treatments, including oral and topical NSAIDs and intra-articular glucocorticoid injections, provide limited relief and functional improvement for only a small subset of patients suffering with KOA [6,7, 11]. Numerous nonsurgical therapies have been recommended to treat pain due to KOA, but all demonstrate only minimal clinically significant improvement [11]. Despite multiple guidelines advising against the chronic use of opioid analgesics, primary care providers often rely on chronic opioid therapy to manage KOA pain, leading to a high rate of opioid use in this population [12,13]. One large database review found that 40 % of patients with KOA were taking opioids [14]. Another review reported that approximately 30 % of KOA patients used opioids, with 9 % reported to be chronic users and 21 % reported to be occasional users [15]. To reduce reliance on opioids many prescribers advocate for either combination treatment, or a stepwise approach, similar to that used in the Veteran’s Health Administration [16,17]. However, none of these have been thoroughly evaluated in large-scale clinical trials.

Both CBT and duloxetine are evidence-based interventions recommended in some clinical guidelines for KOA, although they are not universally adopted in clinical practice [6]. Several reviews of the conservative KOA treatments have found small effect sizes for psychological treatments, such as cognitive-behavioral therapy (CBT) [18,19], and for non-opioid pharmacotherapy with duloxetine [20]. Given the high rate of opioid use in individuals with KOA [13,21], and the high rate of persistent pain after joint replacement, there is a critical need to promote evidence-based interventions that pose fewer risks, offer greater cost-effectiveness, and may provide superior efficacy than opioids and knee arthroplasty.

In Phase 1 of this two-phase pragmatic effectiveness trial, we seek to compare guideline-recommended KOA treatments and determine the comparative effectiveness of conservative, non-opioid pharmacological interventions in improving pain management and function for people with KOA. This study has the potential to influence future treatment guidelines by providing rigorously evaluated, effective non-opioid alternatives. Phase 2 will assess procedural interventions, including intra-articular injection, genicular nerve blocks or ablation, in a broader group of participants—including individuals ineligible for Phase 1 as well as those who express a preference for further treatment—thereby extending the SKOAP trial’s evaluation across a more inclusive range of clinical presentations and treatment pathways. Once completed, the Sequenced strategy for improving outcomes in people with Knee OsteoArthritis Pain (SKOAP) trial will be the most comprehensive evaluation of KOA treatment options to date.

## Methods

### Study design

**Fig. 1.**
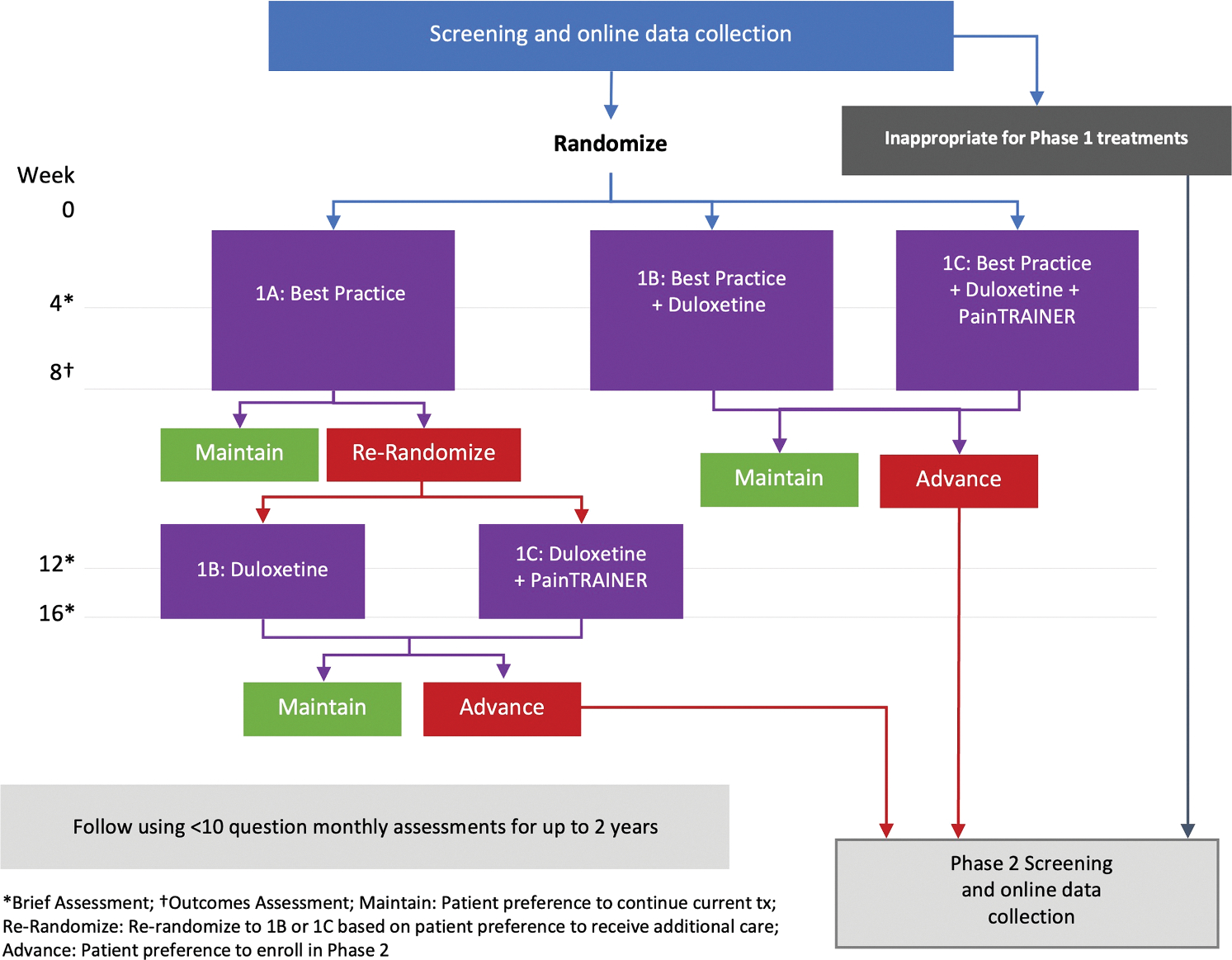
SKOAP phase 1 trial design.

The SKOAP study is a multi-site, randomized, pragmatic, comparative-effectiveness trial with a two-phase sequential design to assess interventions in participants with KOA (ClinicalTrials.gov, NCT04504812). Phase 1 evaluates three non-invasive treatment paradigms in up to 900 participants (see Fig. 1 for an illustration of the study design). Participants are randomized at the baseline visit. Randomization is stratified by site and Knee OA Pain Index (KOAPI) [22] pain level (low <7/10 vs. high ≥7).

### Study setting

Thirty clinical trial sites across the United States are participating in the study, including three Veteran’s Administration (VA) hospitals, one military treatment facility (MTF) and twenty academic medical centers (Supplementary Table 1). The trial is funded through the National Institutes of Health (NIH), Helping to End Addiction Long-term^®^ Initiative (NIH HEAL), Pain Management Effectiveness Research Network (ERN) initiative, via the National Institute of Arthritis, Musculoskeletal and Skin Diseases (NIAMS), the National Institute of Neurological Disorders and Stroke (NINDS) and administered through the National Center for Advancing Translational Science (NCATS) Trial Innovation Network (UH3 AR077360).

### Participants

The full list of inclusion and exclusion criteria is presented in Supplementary Table 2. In brief, eligibility requires meeting one of the three American College of Rheumatology Classification criteria for KOA [12] and having twice reported a knee pain score > 4 and ≤9 on the KOAPI (range: 0–10) [22]. The KOAPI is a combination of 3 items from the Brief Pain Inventory (BPI) pain severity subscale (current, average, and worst pain over the past 7 days) and 1 item from the BPI pain interference subscale (pain interference upon walking in the past 7 days) [22]. General exclusion criteria include: age 〈18 years, pain < 4 days per week over the past 3 months, index knee pain from a joint disease other than OA, changes in analgesic medication dose within 2 weeks of baseline, oral morphine equivalent dose 〉 90 mg/d, severe vision, hearing or cognitive impairment preventing comprehension of consent and study procedures, poorly controlled serious psychiatric condition, history of joint replacement (total knee arthroplasty [TKA]) in the study knee, and pregnancy. There are also Phase 1-specific exclusion criteria, and patients excluded due to these criteria are offered participation and enrollment into Phase 2 of the study. The Phase 1-specific exclusion criteria are known allergic reaction or medical condition prohibiting use of duloxetine; reported failed trial of an adequate dose of duloxetine to relieve KOA symptoms; unreliable internet access; and refusal to participate in Phase 1 combined with having tried and failed ≥2 Best Practices (BP) treatments which are defined below. Trial enrollment began February 2, 2021.

### Interventions

Participants are randomized equally to one of three conservative treatment arms: Best Practices (Arm 1A); BP plus duloxetine (Arm 1B); and BP plus duloxetine plus painTRAINER (Arm 1C). Participants in Arms 1B and 1C are scheduled to receive a call 2 weeks post-randomization to determine whether the duloxetine prescription was filled and to titrate the dose if indicated. There are two follow-up visits scheduled for 4 and 8 weeks (operationalized as 30 and 60 days, respectively) after randomization. At the 8-week visit, outcomes are to be collected, and participants are asked if they want additional treatment. Participants originally assigned to Arm 1B or 1C who express preference for additional treatment will enter the Phase 2 of the study if eligible. Participants from Arm 1A who express a preference for additional conservative treatment will be re-randomized to receive 1B or 1C, unless eligibility criteria dictate that they transition directly to Phase 2. Participants expressing a preference to maintain Phase 1 treatment(s) will complete monthly assessments for up to two years, or until they express preference for additional treatment, or until the trial ends. Data from participants re-randomized into Arms 1B and 1C will not be included in the Phase 1 primary analyses; these data will be incorporated into subsequent secondary analyses as appropriate. All interventions are standard components of routine clinical care and generally covered by insurance. To support equitable access, site staff received centralized training and guidance to help participants select feasible Best Practice options based on clinical profile, patient preference, and potential cost considerations.

### Phase 1A: Best practices

Best Practices (BP), which serves as the Phase 1 control group, includes guideline-recommended treatments (at the time of study initiation) [6] tailored to the patient. The BP menu was created by the Phase 1 Clinical Protocol Committee (Supplementary Table 3). Adherence to BP includes participation in one physical therapy modality *and* at least one alternative approach or use of NSAIDs or other over-the-counter medication. BP options are standardized across sites and documented. Although we anticipate minor variations (e.g., acupuncture or Tai Chi may not be available for all study participants), study clinicians will be trained to review a list of Best Practice options with all Phase 1 participants and collaborate in making choices based on the patient’s interests, clinical profile, and associated costs.

### Phase 1B: Best practices + duloxetine

Best Practices + duloxetine (concurrent use) includes the BP described above and duloxetine, a serotonin and norepinephrine reuptake inhibitor with an approved FDA indication for chronic musculoskeletal pain, as well as other conditions such as depression, anxiety, peripheral diabetic neuropathy, and fibromyalgia. A duloxetine prescription is provided at the Phase 1 baseline visit and study coordinator or clinic staff contact the participant at two weeks post-randomization to confirm whether the prescription was filled and to assess their titration needs. Duloxetine is titrated to a target dose between 30 mg/d to 120 mg/d administered either as a single daily dose or in a twice-per-day dosing regimen, at the discretion of the treating clinician depending on patient-specific variables. The titration period is individualized (i.e., dependent on age, weight, and concomitant medications), with previous studies showing good tolerability when duloxetine is titrated up by 30 mg every 5 to 7 days up to 120 mg [23], a dose range consistent with those used in clinical trials [24].

### Phase 1C: Best practices + duloxetine + painTRAINER

Best Practices + duloxetine + painTRAINER (concurrent use) includes BP and duloxetine as described above, and adds painTRAINER^®^ [25], an 8-week enhanced web-based and self-completed CBT-informed pain coping skills training that includes a manual, skills training, homework/practice assignments and is facilitated, in this study, by a centralized contact. The facilitator position is designed for a research assistant with an undergraduate degree and without training as a clinical psychologist or therapist. The facilitator receives motivational interviewing training and follows a protocolized schedule for contacting participants. They actively support program engagement by encouraging participants to register and by following up with those who have not signed in within a reasonable timeframe and/or are not logging in at regular intervals. Participants are also provided with a written painTRAINER workbook along with optional reminders sent to the patient via email.

### Rescue therapies

Patients are strongly encouraged to avoid starting new medications or increasing their dose of stable medications, though conservative measures such as exercise and weight loss may be continued. For patients requiring rescue therapy, up to 100 mg/day of tramadol (or its oral morphine equivalent for those with a contraindication to tramadol) will be permitted.

### Randomization

Participants are randomized in a 1:1:1 ratio to the three intervention groups. Randomization is determined via permuted blocks and stratified by site and KOAPI (i.e., low (< 7) vs. high (≥ 7)).

### End-of-Study definition

Participants will be followed on a monthly basis for up to two years following their main outcome visit at 8 weeks post-randomization. The trial will be considered complete once the last enrolled participant’s main outcome visit has occurred. The duration of the post-main outcome visit monthly follow-ups will be variable, with some participants completing less than the full 2 years of monthly follow-ups due to their own desire to discontinue the follow-ups, transition into Phase 2 or due to end of study. Participants who discontinue participation in the study treatment protocol are encouraged to continue participation in the main outcome visits and monthly follow-ups, as appropriate.

### Treatment adherence

Adherence to BP treatments is determined by patient self-report at 4- and 8-weeks follow-up visits. Duloxetine adherence is evaluated via self-reported use of the medication and the Medication Adherence Report Scale (MARS-5). The painTRAINER program has built-in software that tracks participation. For each treatment arm, per protocol (PP) treatment adherence will be defined in two ways: receipt of prescription (PP-ROP) and minimal effective dose (PP-MinED).

The PP-ROP definition for Best Practices (1A) requires a prescription for PT, and a prescription for an alternative approach and/or OTC medications; the PP-ROP definition for Arm 1B requires the criteria for Arm 1A plus receiving a duloxetine prescription; the PP-ROP definition for Arm 1C requires the criteria for Arm 1B plus receipt of the painTRAINER workbook with registration information. See Supplementary Table 4 for detailed descriptions. These criteria must be met within 30 days of randomization.

The PP-MinED definition for Arm 1A requires receipt of physical therapy plus an alternative treatment or treatment with a medication from a specified set; PP-MinED for Arm 1B requires the criteria for Arm 1A plus duloxetine at 30 mg for at least 1 week; the PP-MinED for 1C requires the criteria for Arm 1B plus completion of 6 sessions of painTRAINER. See Supplementary Table 5 for detailed descriptions. The PP-MinED criteria for each arm must be completed within 67 days of randomization. Consideration was given to the risk of low adherence due to differences in intervention application across the 30 participating clinical sites. While balancing the importance of pragmatism, the research team provided structure for the sites with an in-depth intervention manual, training materials, manuscripts and provider trainings that included scripts for Phase 1 interventions, as well as the requirement of study staff and providers at each site to take the appropriate training module and pass post-training quizzes. Sites are monitored for protocol adherence, with remedial training to be provided for site personnel who deviate from the established protocol. Every effort is made to standardize implementation across sites, including providing a menu of preferred treatment options for best practices, a recommended titration schedule for duloxetine, and centralized facilitation of the painTRAINER program to ensure consistency in delivery. Additional measures to support fidelity included mandatory training completion with knowledge checks for all site personnel, continuous site monitoring through case report form review, regularly scheduled monthly implementation calls, and structured remediation procedures for sites demonstrating protocol drift.

### Outcomes

The primary outcome is the change in KOAPI (modified 4-Item BPI Pain Scale score) from baseline (randomization) visit to 8 weeks. Secondary efficacy outcome measures will include change in BPI Pain Severity, change in BPI Pain Interference, change in each of 3 subscales of the Knee Injury and Osteoarthritis Outcome Score (KOOS): Pain, Activities of Daily Living (ADL), Quality of Life (QOL), and change in the KOOS Impact Score. Other outcomes will be reported in secondary manuscripts. Outcomes are scheduled to be collected 8 weeks after randomization. See Table 1 for a summary of the study’s measures and which NIH Common Data Elements were collected [26].

### Analysis populations

In a large trial like SKOAP, it is expected that some randomized individuals will be inappropriately enrolled (“inappropriate enrollments”), deemed ineligible based on information acquired post-randomization (“late ineligibles”), or administratively withdrawn (“administrative withdrawals”). These individuals will be excluded from the primary analyses, which will be conducted using the modified intention-to-treat (mITT) population. Administrative withdrawals occurred following the planned interim analysis that led to the closure of Arm 1A; participating sites were notified of this closure on 11/29/2023. Specifically, individuals randomized to Arm 1A after 9/30/2023 were administratively withdrawn, as they had no opportunity for primary outcome assessment. Follow-up time for all Arm 1A participants is censored at 11/29/2023. To allow for valid comparisons between Arms 1A and 1B, the Arm 1B population will be censored at the same timepoint. Main outcome papers will report results for both Arm 1B subgroups: one censored for comparison to 1A, and the full sample for comparison to Arm 1C. Whenever possible, all randomized participants will continue to be followed to collect outcome and adverse event data. The safety population, used for evaluating serious adverse events (SAEs) and adverse events (AEs), will include all randomized participants who were exposed to any study intervention.

### Adjudication

An independent adjudication process will be developed and a committee with relevant expertise will be assembled. Blinded to treatment assignment, the committee will adjudicate inappropriate enrollments, late ineligibles and identify participants meeting the criteria for PP-ROP and PP-MinED for Best Practices. The committee will be partially blinded regarding classification of PP-ROP and PP-MinED for arms including duloxetine; it will be unblinded for PP-ROP and PP-MinED classification of painTRAINER. All decisions by this committee will be by majority rule.

### Estimands

The mITT estimand quantifies the effect of treatment assignment in the mITT population. The Receipt of Prescription (PP-ROP) estimand quantifies the effect of receiving access to the treatments associated with the assigned components in the mITT population (i.e., the effect if everyone in the mITT population received each of the assigned prescriptions). The PP-MinED estimand quantifies the effect of adherence to the minimal effective dose associated with the assigned treatments in the mITT population (i.e., the effect if everyone in the mITT population received at least the minimal effective dose for each component of treatment arms).

### Power considerations and interim analysis

The trial is powered to address mITT effects. Specifically, it is powered to answer the following two questions:

1. Is there a difference in KOAPI 8-week change score between assignment to Best Practices + duloxetine (1B) versus assignment to best practices (1A) arms?
2. Is there a difference in KOAPI 8-week change score between assignment to Best Practices + duloxetine + painTRAINER (1C) versus assignment to Best Practices + duloxetine (1B) arms?

The sample size calculation is based on the following assumptions: (a) overall type I error preserved at 5 %, (b) effect sizes of 0.3 for each of the contrasts, (c) 10 % missing outcome data and (d) one interim analysis after half the patients have enrolled and reached 8 weeks of follow-up. A conservative Bonferroni correction will be applied so that the type 1 error for each contrast is set to 2.5 %. Table 2 specifies the interim analysis stopping/continuation rules. With an enrollment sample size of 900 participants, the design has the following operating characteristics (assessed by simulation using pairwise *t*-tests):

- Greater than 88.4 % chance of declaring superiority of 1B over 1A
- Greater than 91.1 % chance of declaring superiority of 1C over 1B
- 17.6 % chance of stopping 1A at the interim analysis (Scenario 3)
- 0.82 % chance of stopping early (Scenarios 1, 2, 4, 5, 6)

### Statistical approach

Our mITT analysis will (1) leverage baseline covariates, (2) account for off-schedule assessment times and (3) account for loss to follow-up. Three baseline covariates will be leveraged: KOAPI, chronic opioid use and diffuse pain phenotype score. For secondary outcomes, the baseline value of the outcome will also be included as a leveraging covariate. We will use an augmented inverse weighting approach to estimate, for each treatment group, the mean change from randomization in KOAPI (and secondary outcomes), modeled as a function of time since randomization; treatment effects will be evaluated at the median assessment time, pooled across treatment groups. For the primary outcome, each contrast will involve construction of a two-sided 97.58 % confidence interval (reflecting type 1 error spent at the interim analysis). If the confidence interval excludes zero, superiority/inferiority of the relevant treatment arm will be declared. For the secondary outcomes, each contrast will involve construction of a two-sided 97.5 % confidence interval.

Our PP-ROP and PP-MinED analyses will (1) adjust for baseline confounding factors, (2) account for off-schedule assessment times, and (3) account for loss to follow-up. To the extent possible, baseline factors will include: KOAPI, chronic opioid use, diffuse pain phenotype score, baseline value of the outcome (for secondary outcome change scores), self-efficacy for disease management, internet access, resiliency, catastrophizing, age, and sex assigned at birth. We will use an augmented inverse weighting approach to estimate, for each treatment group, the mean change from randomization in KOAPI (and secondary outcomes) as a function of time since randomization. For PP-ROP, treatment effects will be evaluated at the median time of assessment for the participants who followed the protocol; for the PP-MinED, treatment effects will be evaluated at the maximum of 67 days and the median time of assessment for the patients who followed the minimal effective dose. For each outcome and each contrast, a two-sided 97.5 % confidence interval will be reported.

## Discussion

Effective nonsurgical treatments for KOA pain are essential to reduce reliance on opioids and provide meaningful options for patients who are unable or unwilling to undergo joint replacement surgery. While a range of nonsurgical treatments exist, large-scale randomized trials are necessary to generate consistent, evidence-based treatment guidelines for the management of KOA.

This study aims to evaluate the effectiveness of commonly recommended non-opioid treatments, alone and in combination, for improving pain and function in individuals with KOA. Additionally, it seeks to identify clinical and patient-level factors associated with treatment response, with the aim of informing more personalized and targeted care strategies. If proven to be beneficial over commonly prescribed standard treatments, duloxetine and/or painTRAINER treatment approaches could be prioritized earlier in the treatment pathway, potentially reducing the need for more invasive procedures such as injections or surgery. In doing so, this approach may help delay or prevent knee replacement surgery, reducing healthcare costs and minimizing the risks associated with total knee arthroplasty (TKA) [10,27].

A key strength of this study is its real-world approach to evaluating KOA treatments using therapies already in use in pain management practice. The multi-site randomized comparative effectiveness design enhances the study’s diverse patient populations, clinical environments, and healthcare systems. By including a range of clinical settings—VA hospitals, military treatment facilities, and academic medical centers across geographic locations—the study ensures broader relevance and has the potential to generate insights applicable to a wide spectrum of KOA patients.

Furthermore, the study’s use of both combined and sequential treatment approaches reflects real-world clinical decision-making, allowing multidisciplinary therapies to be tailored based on patient response and introducing new treatments in a stepwise fashion. This design ensures participants receive evidence-based multimodal care while also helping to identify the most effective conservative treatments. The emphasis on patient-centered outcomes, such as reductions in pain, functional improvement, and quality of life, reinforces the study’s clinical relevance and its potential to inform standard medical practice. This foundation informs Phase 2 of the SKOAP trial, which will evaluate procedural interventions in a broader population—including those ineligible for or unsatisfied with Phase 1 treatments.

Finally, recent research in both spine and knee osteoarthritis populations has highlighted the value of combining CBT with structured exercise. In KOA-specific studies, a recent meta-analysis found that physical therapist-delivered CBT combined with exercise led to significant reductions in pain scores, both for in-person and remote delivery formats [28]. However, this meta-analysis was based on a small number of trials and demonstrated moderate to high heterogeneity and should therefore be interpreted with caution. Similarly, a multicenter feasibility trial demonstrated the practical integration of internet-delivered CBT into preoperative KOA rehabilitation pathways [29]. These findings parallel evidence from spine surgery studies, where structured exercise plus CBT outperformed physical therapy alone [30,31], and support a growing shift toward multimodal, patient-centered approaches in chronic musculoskeletal pain management.

Despite its strengths, the SKOAP trial also faces challenges. Adherence to outpatient treatment protocols may vary widely, affecting engagement and potential efficacy. To address this, the study will implement structured follow-ups and digital engagement strategies to improve adherence. Additionally, patient dropout over time is a concern, as KOA is a chronic condition, and some participants may not experience immediate benefits. Proactive communication and structured follow-ups will be used to enhance retention. Some treatments are not covered by available payors and thus may preclude some patients from Phase 1 treatments, though reduced cost options are available for best practices, duloxetine/generic is relatively inexpensive, and painTRAINER was offered free of charge. Although these low-cost and broadly available treatments were prioritized, access barriers may still affect participation among lower-resourced populations and introduce disparities in engagement.A further concern in this regard is the fact that no widely accepted definition of per protocol exists for these treatments. While several other definitions could have been used, the study team settled on two: receipt of prescription (PP-ROP) and minimal effective dose (PP-MinED). The lack of a single standard definition may contribute uncertainty to the results. Moreover, although combining conservative treatments may improve outcomes for some subgroups, their average individual effects remain modest. Thus, even in combination, population-level treatment effects may be limited, and this possibility should be considered when interpreting results.

Another limitation is that participants cannot be blinded to their treatment group, which may introduce expectation bias. To minimize this, several strategies were implemented, including blinded data analysis, direct self-report of outcomes into the electronic data capture system (EDC), and a partially blinded adjudication process. Also, research staff were trained to interact with subjects to describe the three treatment arms as impartially as possible. Additionally, while the trial provides valuable short- and intermediate-term data, the long-term effectiveness of these treatments remains uncertain. Future research, including long-term follow-up studies, will be necessary to evaluate the lasting impact of these non-opioid treatments on pain management and opioid reduction. As specified in the protocol, participants are followed on a monthly basis for up to two years post-randomization unless they withdraw or enter Phase 2. These longitudinal effects will be examined in secondary analyses to explore the durability of treatment response over time.

The findings from SKOAP will inform evidence-based, personalized treatment strategies for KOA pain management, potentially reducing reliance on opioids. By identifying key clinical and psychosocial factors that influence treatment response, this research may pave the way for more effective, patient-centered approaches to long-term reductions in pain.

## Supplementary Material

Supplementary Material

Supplementary materials

Supplementary material associated with this article can be found, in the online version, at doi:10.1016/j.semarthrit.2025.152834.

## Figures and Tables

**Table 1:** SKOAP measures.

Domain	Measure	Common Data Element

Pain	BPI Severity & Interference (including KOAPI†) Knee Injury and Osteoarthritis Outcome Score (KOOS) short form High Impact Chronic Pain Widespread Pain Inventory/Symptom Severity Central Sensitization Inventory Brief General Sensory Sensitivity Screen	X
Physical Function/Quality of Life	PROMIS Physical Functioning Short Form	X
Fatigue	PROMIS Fatigue	
Sleep	PROMIS Sleep Disturbance + Sleep Duration	X
Pain Coping	Pain Catastrophizing Scale – Short Form Chronic Pain Coping Scale, Short form	X
Depression	PHQ-8 (includes PHQ-2 for CDE)	X
Anxiety	GAD-7 (includes GAD-2 for CDE)	X
Adverse Childhood Experiences	BRFSS Screen	
Social Determinants of Health	Social Determinants Screen	
PTSD	PTSD Brief Screen	
Substance Use	Tobacco, Alcohol, Prescription medications, and other Substance (TAPS)	X
Resilience	Connor-Davidson Resilience Scale (CD-RISC)	
Self-Efficacy	ASES8	
Treatment Motivation	Pain Relief Motivational Scales – Short Form	
Treatment Satisfaction	Patient Global Impression of Change (PGIC)	X
Treatment Adherence	MARS5	

KOAPI†=primary outcome.

**Table 2:** Interim analysis action plan.

Scenario	BP+*D* vs. BP (1B vs. 1A)	BP+*D*+CBT vs. BP+*D* (1C vs. 1B)	Action

1	BP+*D* superior	BP+*D*+CBT superior	STOP PHASE 1
2	BP+*D* superior	BP+*D* superior	STOP PHASE 1
3	BP+*D* superior	Insufficient evidence	STOP BP
4	BP superior	BP+*D*+CBT superior	STOP PHASE 1
5	BP superior	BP+*D* superior	STOP PHASE 1
6	BP superior	Insufficient evidence	STOP PHASE 1
7	Insufficient evidence	BP+*D*+CBT superior	CONTINUE
8	Insufficient evidence	BP+*D* superior	STOP BP+*D*+CBT
9	Insufficient evidence	Insufficient evidence	CONTINUE
