# Supplementary material for: Study design and protocol of a randomized, pragmatic, comparative effectiveness trial evaluating a sequenced strategy for improving outcomes in people with knee osteoarthritis pain (SKOAP): Conservative treatment evaluation
Source: Semin Arthritis Rheum. Author manuscript; Available in PMC 2026 Jan 5. (PMC12766632; doi:10.1016/j.semarthrit.2025.152834)
Supplement: Supplementary Material [file NIHMS2116766-supplement-Supplementary_Material.docx]

**Study design and protocol of a randomized, pragmatic, comparative effectiveness trial evaluating a sequenced strategy for improving outcomes in people with knee osteoarthritis pain (SKOAP): Conservative treatment evaluation**

**Supplemental Tables**

## Supplemental Table 1. SKOAP Sites

| **Site** | **Activation Date** | **Closure Date** |
| --- | --- | --- |
| UUT | 1/19/2021 |  |
| JHU | 1/20/2021 |  |
| UCS | 1/21/2021 |  |
| UMD | 1/21/2021 | 3/21/2023 |
| HBW | 1/22/2021 |  |
| WFU | 2/1/2021 |  |
| UVA | 2/18/2021 |  |
| OHS | 2/25/2021 |  |
| MIN | 3/10/2021 |  |
| UWA | 3/10/2021 |  |
| UFL | 3/18/2021 |  |
| CBU | 3/23/2021 | 4/10/2023 |
| VMC | 4/20/2021 |  |
| WCM | 5/12/2021 |  |
| NWU | 5/19/2021 |  |
| CVA | 5/3/2022 |  |
| EMU | 5/9/2022 |  |
| PVA | 6/23/2022 | 11/13/2023 |
| COL | 7/1/2022 |  |
| UIA | 7/12/2022 |  |
| UAB | 7/12/2022 | 11/9/2023 |
| UAR | 7/22/2022 | 11/27/2023 |
| UCD | 9/8/2022 |  |
| UNC | 9/29/2022 |  |
| CLE | 12/16/2022 |  |
| MAY | 4/12/2023 | 11/8/2023 |
| ROC | 10/24/2023 | 8/2/2024 |
| WRD | 10/30/2023 |  |
| AVA | 11/1/2023 |  |
| SVA | 11/3/2023 |  |

## Supplemental Table 2. Participant Inclusion/Exclusion Criteria

**Inclusion Criteria:** To be eligible to participate in this study, an individual must meet one of the three American College of Rheumatology Classification criteria for KOA and have knee pain. Study-eligible knee pain and detailed descriptions of the ACR criteria sets are provided in the table below.

| Knee Pain score of ≥4 and ≤ 9 on the KOAPI (Modified 4-Item BPI Pain Scale*) (0-10 point scale) at pre-intervention screening;  ***AND*** | | |
| --- | --- | --- |
| *At least 3 of the following using* ***history and physical examination*** | *At least 1 of the following using* ***history, physical examination, and radiographic findings + the presence of osteophytes*** | *At least 5 of the following using* ***history, physical examination, and laboratory findings*** |
| - Age > 50 years old - Morning stiffness < 30 minutes - Crepitus on knee motion - Bony tenderness - Bony enlargement - No palpable warmth | - Age > 50 years old - Morning stiffness < 30 minutes - Crepitus on active motion and osteophytes | - Age > 50 years old - Morning stiffness < 30 minutes - Crepitus on knee motion - Bony tenderness - Bony enlargement - No palpable warmth - ESR < 40 mm/hour - Rheumatoid Factor (RF) < 1:40 - Synovial fluid signs of osteoarthritis |
| - The KOAPI, a 4-Item modification of the BPI Pain Scale is a combination of 3 items from the BPI Pain Intensity subscale and 1 item from the BPI Pain Interference subscale. The pain intensity items ask about average pain, current pain, and worst pain in the past 7 days. The pain interference item asks about pain interference upon walking in the past 7 days. | | |

**Exclusion Criteria:**

General Exclusion Criteria

| An individual who meets any of the following criteria will be excluded from participation in this study:   - Aged below 18 years - Any inability to complete study procedures, including, but not limited to inadequate resources to mitigate low English language literacy - Refusal of randomization - Knee Pain Exclusions:   - Pain during an average of < 4 days per week over the past 3 months   - Pain in the index knee from a joint disease other than OA (e.g., infectious arthritis, rheumatoid arthritis, spondyloarthropathy) - Medication Exclusions:   - Report changes in analgesic medication dose within 2 weeks of baseline   - Oral morphine equivalent dose of > 90 mg/d at baseline - Medical Condition Exclusions:   - Severe vision or hearing impairment or any signs of cognitive impairment that would prevent comprehension of consent procedures, study measures, or procedures.   - Unstable medical condition that presents an absolute or relative contraindication for participation in both arms (e.g., unstable angina, congestive heart failure).   - Poorly controlled serious psychiatric condition that could prevent full participation or affect outcomes (e.g., suicidal ideation, active psychosis, poorly controlled depression, active substance abuse [excluding tobacco, caffeine, or moderate alcohol use]) - Knee‑specific Medical Condition Exclusions:   - History of bilateral knee joint replacement arthroplasty (total knee arthroplasty [TKA]) or TKA in the affected knee; partial replacements may be eligible depending on physician judgment   - Scheduled joint replacement   - History of unilateral TKA and complaints of KOA pain limited to the operated knee   - Intra-articular viscosupplementation, steroid injection or arthroscopic surgery in the index knee within 12 weeks of baseline - Pregnancy by self-report/report of intention to become pregnant (Phase 1) or by urine pregnancy screening (if SOC at site) (Phase 2). Due to the unknown effects of duloxetine on the developing fetus and newborn, and the potential harms of fluoroscopy in pregnancy, women who are pregnant or lactating or intend to get pregnant will not be included in this study. Those of childbearing potential will be asked to use reliable contraception during the course of their participation in the study and to notify the study team if they become pregnant during participation. Definition of reliable birth control will be defined as:   - Female and male sterilization (female tubal ligation or occlusion, male vasectomy)   - Long-acting reversible contraceptives or "LARC" methods (intrauterine devices, hormonal implants)   - Short-acting hormonal methods (pill, mini pills, patch, shot, vaginal ring)   - Barrier methods (condoms, diaphragms, sponge, cervical cap) |
| --- |

Phase 1‑specific Exclusion Criteria

| An individual who meets any of the following criteria will be excluded from participation in Phase 1 of this study and will be enrolled and randomized directly into Phase 2:   - Known allergic reaction or medical condition that renders an individual unsuitable for Phase 1 study interventions, including closed-angle glaucoma, kidney disease (creatinine clearance < 30 mL/ min), severe liver disease, known adverse reaction to duloxetine or another selective serotonin-norepinephrine reuptake inhibitor (SNRI), bipolar disorder or mania, high likelihood of drug interactions that could lead to side effects (e.g., serotonin syndrome in people on multiple drugs that inhibit serotonin reuptake including monoamine oxidase [MAO] inhibitors). - Report failed trial of an adequate dose of duloxetine to relieve KOA symptoms over a 1-month period - Have tried and failed two of the following: NSAIDs, physical therapy (there are many physical therapies so clinicians should exercise their judgment as to what constitutes ‘failed’ therapy), or weight loss (need determined by clinician) and refuses participation in Phase 1 - End-stage renal disease - Unreliable access to the internet on a daily basis^1^     ^1^Reliable daily access to the internet, defined as sufficient access to participate in the study and may include public library access, café/ coffee shop access, access to a friend/neighbor’s Wi-Fi or hotspot, etc. Reliability will be determined on a site-by-site basis.  Note that Phase 1 solo participants that initially were not interested in Phase 2 and change their mind and are eligible for Phase 2, may transition. |
| --- |

**Supplemental Table 3. Best Practices Menu. Completed at Phase 1 Baseline session by provider collaboratively with study participants.**

Select one or more options from the ‘Physical Therapies’ group and at least one option from the ‘Alternative Approaches’ or ‘Medication’ groups. The preferred treatment is underlined, but if the participant has already tried that, select a different practice that they are interested in and you think would benefit them. Please circle the programs they agree to try for the next eight weeks and record the regimen you are suggesting.

|  | *One from this column:* | *At least one from either of these two columns:* | |
| --- | --- | --- | --- |
|  | **Physical Therapies** | **Alternative Approaches** | **Medication** |
| Preferred: | - Physical Therapy   *# of sessions*  *per week: _______* | - Weight management   (if appropriate/BMI >30;  circle your recommendation)     - - Weight loss program   - Calorie reduction diary   - Bariatric surgery   - Support groups   - Diet pills   - Exercise | - NSAIDs   *Name of drug _____________*  *Circle: Topical / oral*  *Dose _____________*  *Times / day _____________*  *Or as needed*    *Name of drug _____________*  *Circle: Topical / oral*  *Dose _____________*  *Times / day _____________*  *Or as needed* |
|  | - Exercise program   (including water aerobics)  *# of sessions*  *per week: _______* | - Acupuncture - Meditation - Yoga - Tai chi - Other:   _______________________  _______________________  _______________________ | - Topical capsaicin - Acetaminophen - Other _______­­­___________   __________________  *Name of drug _____________*  *Dose _____________*  *Times / day _____________*  *Or as needed*  *Name of drug _____________*  *Dose _____________*  *Times / day _____________*  *Or as needed*  *Name of drug _____________*  *Dose _____________*  *Times / day _____________*  *Or as needed* |
|  | - Biomechanical   (circle one)   - - Knee braces   - Canes   - Kinesiology tape     *# per week: _______*    *Or as needed* |  |  |
|  | - Therapeutic Heating/Cooling   *# per week: _______*    *Or as needed* |  |  |
|  | Other_________  *# per week: _______*    *Or as needed* |  |  |

**Supplemental Table 4. Per-Protocol, Receipt of Prescription (PP-ROP) Definitions**

| Arms | Assigned Interventions |
| --- | --- |
| Active Comparator: Phase 1A: Best Practices  Participants will receive a prescription for guideline-recommended treatments for knee osteoarthritis, i.e., Best Practices. Best Practices can include topical or oral nonsteroidal anti-inflammatory drugs (NSAIDs), acetaminophen; physical therapy that may include aquatherapy; alternative approaches such as acupuncture, yoga, or a structured exercise program; and other non-invasive treatments.  Operationally: A prescription for PT, alternative approaches, or OTC medications | Best Practices  Best Practices can include topical or oral nonsteroidal anti-inflammatory drugs (NSAIDs), acetaminophen; physical therapy that may include aquatherapy; alternative approaches such as acupuncture, yoga, or a structured exercise program; and other non-invasive treatments. |
| Active Comparator: Phase 1B: Best Practices + Duloxetine  Participants will receive Duloxetine and a prescription for guideline-recommended treatments for knee osteoarthritis, i.e., Best Practices. Best Practices can include topical or oral nonsteroidal anti-inflammatory drugs (NSAIDs), acetaminophen; physical therapy that may include aquatherapy; alternative approaches such as acupuncture, yoga, or a structured exercise program; and other non-invasive treatments.  Operationally: Adherence to arm 1A plus receiving and filling a duloxetine prescription | Drug: Duloxetine  Duloxetine is a drug that is used to improve pain and function in people with knee osteoarthritis (KOA). Duloxetine is approved by the Food and Drug Administration (FDA) for the treatment of depression, anxiety disorder, fibromyalgia, and joint pain. It will be titrated up from 20 or 30mg according to a schedule provided by a study provider.  Other Names:   - Cymbalta   Best Practices  Best Practices can include topical or oral nonsteroidal anti-inflammatory drugs (NSAIDs), acetaminophen; physical therapy that may include aquatherapy; alternative approaches such as acupuncture, yoga, or a structured exercise program; and other non-invasive treatments. |
| Active Comparator: Phase 1C: Best Practices + Duloxetine + painTRAINER  Participants will receive Duloxetine, pain coping skills training, and a prescription for guideline-recommended treatments for knee osteoarthritis, i.e., Best Practices. Best Practices can include topical or oral nonsteroidal anti-inflammatory drugs (NSAIDs), acetaminophen; physical therapy that may include aquatherapy; alternative approaches such as acupuncture, yoga, or a structured exercise program; and other non-invasive treatments.  *Note that the term ‘painTRAINER’ is not used in our clinicaltrials.gov description of the study or in any recruitment materials in order to reduce likelihood that patients not enrolled in this arm would find/participate in this publicly available resource.  Operationally: Adherence to Arms 1A and 1B plus receipt of the painTRAINER workbook | Drug: Duloxetine  Duloxetine is a drug that is used to improve pain and function in people with knee osteoarthritis (KOA). Duloxetine is approved by the Food and Drug Administration (FDA) for the treatment of depression, anxiety disorder, fibromyalgia, and joint pain. It will be titrated up from 20 or 30mg according to a schedule provided by a study provider.  Other Names:   - Cymbalta   Behavioral: Pain Coping Skills Training  Participants will be provided with a written manual that includes login information for the pain coping skills training website. The participants will be expected to log into the system weekly, work through the modules, and participate in skills practice. This intervention will be conducted in combination with best practices and duloxetine.  Best Practices  Best Practices can include topical or oral nonsteroidal anti-inflammatory drugs (NSAIDs), acetaminophen; physical therapy that may include aquatherapy; alternative approaches such as acupuncture, yoga, or a structured exercise program; and other non-invasive treatments. |

**Supplemental Table 5. Per-Protocol, Minimum Effective Dose (PP-MinED) Definitions**

| **Arm** |  | **Specific Treatments** | **PP-MinED Definitions. Receipt of below (over a 4-week period):** |
| --- | --- | --- | --- |
| **1A.** **Best Practices** | **Physical Therapies** | Physical Therapy (PT) | >3 sessions of PT OR 1 PT session plus 3 exercise sessions[1-3] |
|  |  | Exercise | >12 sessions[1, 2, 4-7] |
|  |  | Biomechanical  Knee Braces  Canes  Kinesiology tape | >0%[1, 2, 8-12] |
|  |  | Therapeutic Heating/Cooling | >0 uses[1, 13] |
|  |  | Balance Training | >12 sessions[1, 2] |
|  |  | Other | >12 sessions (additive and inclusive of all appropriate treatments in Physical Therapies category) |
|  | **Alternative Approaches** | Weight Management | >5% reduction in body weight from first measurement[1, 2, 14, 15] |
|  |  | -Enrolled in a commercial weight management program | Enrolled in a program |
|  |  | -Followed a specific dietary approach  -Followed their own calorie reduction dietary approach | >80% of meals[16] |
|  |  | -Bariatric Surgery | Had surgery |
|  |  | -Support groups | >3 sessions[17] |
|  |  | -Diet pills | >28 pills[18] |
|  |  | -Exercise | >12 sessions[1, 2, 4-7] |
|  |  | Acupuncture | >4 sessions[1, 2, 19] |
|  |  | Meditation | >5 sessions[1, 2, 20] |
|  |  | Yoga | >8 sessions[1, 2, 21] |
|  |  | Tai chi | >8 sessions[1, 2, 22] |
|  |  | Other | >4 sessions (additive and inclusive of all appropriate treatments in alternative approaches category) |
|  | **Medication** | NSAIDs | >7 doses[1, 2, 23-25] |
|  |  | Topical Capsaicin | >7 doses[1, 2, 23, 26, 27] |
|  |  | Acetaminophen | >7 doses[1, 2, 23, 25, 28] |
|  |  | Other | >7 doses (additive and inclusive of all appropriate treatments in Medication category) |
| **1B. Duloxetine.**  Meet the 1A criteria plus this duloxetine criteria | **Duloxetine** |  | Dose >30 for 1wk+ OR stopped or reduced duloxetine due to AE OR stopped or reduced based on provider advise[29-34] |
| **1C. painTRAINER.**  Meet the 1B criteria plus painTRAINER as defined here. | **painTRAINER** |  | Complete 6 sessions by the outcome visit[35] |

**Supplemental Table 6. Time to Outcome**

The following table contains an overview of the “time zero” point, and the “outcome time” point, which will be used to calculate the changes in KOAPI score for primary analyses.

| Analysis | Estimand is the change in KOAPI between: | |
| --- | --- | --- |
|  | Time 0 | Outcome Time |
| mITT Analysis | Randomization | Median time of first measurement of outcome assessment across all treatment groups. |
| PP-ROP Analysis | Randomization | The target time of assessment will be the median of the observed times of assessment among adherers, pooled over treatment groups. |
| PP-MinED Analysis | Randomization | The target time of assessment will be the median of the observed times of assessment among adherers, pooled over treatment groups. |

Reference List

References of Supplemental tables

1. Brophy, R.H. and Y.A. Fillingham, *AAOS Clinical Practice Guideline Summary: Management of Osteoarthritis of the Knee (Nonarthroplasty), Third Edition.* J Am Acad Orthop Surg, 2022. **30**(9): p. e721-e729.

2. Kolasinski, S.L., et al., *2019 American College of Rheumatology/Arthritis Foundation Guideline for the Management of Osteoarthritis of the Hand, Hip, and Knee.* Arthritis Rheumatol, 2020. **72**(2): p. 220-233.

3. Deyle, G.D., et al., *Effectiveness of manual physical therapy and exercise in osteoarthritis of the knee. A randomized, controlled trial.* Ann Intern Med, 2000. **132**(3): p. 173-81.

4. Guo, X., et al., *A recommended exercise program appropriate for patients with knee osteoarthritis: A systematic review and meta-analysis.* Front Physiol, 2022. **13**: p. 934511.

5. Farr, J.N., et al., *Physical activity levels in patients with early knee osteoarthritis measured by accelerometry.* Arthritis Rheum, 2008. **59**(9): p. 1229-36.

6. Clark, J.E., *The impact of duration on effectiveness of exercise, the implication for periodization of training and goal setting for individuals who are overfat, a meta-analysis.* Biol Sport, 2016. **33**(4): p. 309-333.

7. Del Vecchio, A., et al., *The increase in muscle force after 4 weeks of strength training is mediated by adaptations in motor unit recruitment and rate coding.* J Physiol, 2019. **597**(7): p. 1873-1887.

8. Yu, S.P., et al., *Effectiveness of knee bracing in osteoarthritis: pragmatic trial in a multidisciplinary clinic.* International Journal of Rheumatic Diseases, 2016. **19**(3): p. 279-286.

9. Jones, A., et al., *Impact of cane use on pain, function, general health and energy expenditure during gait in patients with knee osteoarthritis: a randomised controlled trial.* Ann Rheum Dis, 2012. **71**(2): p. 172-9.

10. Abolhasani, M., et al., *Effects of kinesiotaping on knee osteoarthritis: a literature review.* J Exerc Rehabil, 2019. **15**(4): p. 498-503.

11. Cho, H.Y., et al., *Kinesio taping improves pain, range of motion, and proprioception in older patients with knee osteoarthritis: a randomized controlled trial.* Am J Phys Med Rehabil, 2015. **94**(3): p. 192-200.

12. Ogut, H., et al., *Does Kinesiology Taping Improve Muscle Strength and Function in Knee Osteoarthritis? A Single-Blind, Randomized and Controlled Study.* Arch Rheumatol, 2018. **33**(3): p. 335-343.

13. Denegar, C.R., et al., *Preferences for heat, cold, or contrast in patients with knee osteoarthritis affect treatment response.* Clin Interv Aging, 2010. **5**: p. 199-206.

14. Atukorala, I., et al., *Is There a Dose-Response Relationship Between Weight Loss and Symptom Improvement in Persons With Knee Osteoarthritis?* Arthritis Care Res (Hoboken), 2016. **68**(8): p. 1106-14.

15. Kan, H.S., et al., *Non-surgical treatment of knee osteoarthritis.* Hong Kong Med J, 2019. **25**(2): p. 127-133.

16. Hernandez, M., *80/20 Diet Efficacy in Regard to Physiology and Psychosocial Factors.* Journal of Obesity & Weight Loss Therapy, 2017. **07**.

17. Abbott, S., et al., *Group versus one-to-one multi-component lifestyle interventions for weight management: a systematic review and meta-analysis of randomised controlled trials.* Journal of Human Nutrition and Dietetics, 2021. **34**(3): p. 485-493.

18. Jeong, D. and R. Priefer, *Anti-obesity weight loss medications: Short-term and long-term use.* Life Sciences, 2022. **306**: p. 120825.

19. Luo, X., et al., *Acupuncture for treatment of knee osteoarthritis: A clinical practice guideline.* Journal of Evidence-Based Medicine, 2023. **16**(2): p. 237-245.

20. Zautra, A.J., et al., *Comparison of cognitive behavioral and mindfulness meditation interventions on adaptation to rheumatoid arthritis for patients with and without history of recurrent depression.* J Consult Clin Psychol, 2008. **76**(3): p. 408-421.

21. Ghasemi, G.A., A. Golkar, and S.M. Marandi, *Effects of hata yoga on knee osteoarthritis.* Int J Prev Med, 2013. **4**(Suppl 1): p. S133-8.

22. Ye, J., et al., *Mindful Exercise (Baduanjin) as an Adjuvant Treatment for Older Adults (60 Years Old and Over) of Knee Osteoarthritis: A Randomized Controlled Trial.* Evid Based Complement Alternat Med, 2020. **2020**: p. 9869161.

23. Richard, M.J., J.B. Driban, and T.E. McAlindon, *Pharmaceutical treatment of osteoarthritis.* Osteoarthritis Cartilage, 2023. **31**(4): p. 458-466.

24. da Costa, B.R., et al., *Effectiveness of non-steroidal anti-inflammatory drugs for the treatment of pain in knee and hip osteoarthritis: a network meta-analysis.* The Lancet, 2017. **390**(10090): p. e21-e33.

25. Lim, W.B. and O. Al-Dadah, *Conservative treatment of knee osteoarthritis: A review of the literature.* World J Orthop, 2022. **13**(3): p. 212-229.

26. Conley, B., et al., *Core Recommendations for Osteoarthritis Care: A Systematic Review of Clinical Practice Guidelines.* Arthritis Care Res (Hoboken), 2023. **75**(9): p. 1897-1907.

27. Bannuru, R.R., et al., *OARSI guidelines for the non-surgical management of knee, hip, and polyarticular osteoarthritis.* Osteoarthritis Cartilage, 2019. **27**(11): p. 1578-1589.

28. Towheed, T., et al., *Acetaminophen for osteoarthritis.* Cochrane Database of Systematic Reviews, 2006(1).

29. Pritchett, Y.L., et al., *Duloxetine for the management of diabetic peripheral neuropathic pain: response profile.* Pain Med, 2007. **8**(5): p. 397-409.

30. Pritchett, Y.L., et al., *Use of effect size to determine optimal dose of duloxetine in major depressive disorder.* J Psychiatr Res, 2007. **41**(3-4): p. 311-8.

31. Arnold, L.M., S. Zhang, and B.A. Pangallo, *Efficacy and safety of duloxetine 30 mg/d in patients with fibromyalgia: a randomized, double-blind, placebo-controlled study.* Clin J Pain, 2012. **28**(9): p. 775-81.

32. Li, D., et al., *Co-treatment with Oral Duloxetine and Intraarticular Injection of Corticosteroid plus Hyaluronic Acid Reduces Pain in the Treatment of Knee Osteoarthritis.* Pain Physician, 2024. **27**(1): p. E45-E53.

33. Chappell, A.S., et al., *Duloxetine, a centrally acting analgesic, in the treatment of patients with osteoarthritis knee pain: a 13-week, randomized, placebo-controlled trial.* Pain, 2009. **146**(3): p. 253-260.

34. Skljarevski, V., et al., *A double-blind, randomized trial of duloxetine versus placebo in the management of chronic low back pain.* Eur J Neurol, 2009. **16**(9): p. 1041-8.

35. Rini, C., et al., *Automated Internet-based pain coping skills training to manage osteoarthritis pain: a randomized controlled trial.* Pain, 2015. **156**(5): p. 837-848.
